# The NLRP3 inflammasome: molecular activation and regulation in spermatogenesis and male infertility; a systematic review

**DOI:** 10.1186/s12610-022-00157-9

**Published:** 2022-05-30

**Authors:** Marziyeh Tavalaee, Mohsen Rahmani, Joël R. Drevet, Mohammad Hossein Nasr-Esfahani

**Affiliations:** 1 Department of Animal Biotechnology, Reproductive Biomedicine Research Center, Royan Institute for Biotechnology, ACECR, Isfahan, Iran; 2 GReD Institute, Faculty of Medicine, INSERM-CNRS-Université Clermont Auvergne, Clermont-Ferrand, France; 3 Isfahan Fertility and Infertility Center, Isfahan, Iran

**Keywords:** Inflammatory processes, Male fertility, Varicocele, NLRP3, Caspase 1, ASC: apoptosis-associated speck-like protein containing caspase recruitment domain

## Abstract

**Background:**

Infertility related to varicocele, infections, metabolic dysfunctions, oxidative stress and environmental toxicants is also associated with inflammatory processes that ultimately lead to the activation of the inflammasome pathway (IP). IP is classically activated by DAMPs, MAMPs or LAMPs, which stand for Damage-, Microbe- or Lifestyle-Associated Molecular Patterns, respectively. The most important player in IP activation is the NLRP3 (NOD[Nuclear oligomerization domain]-, LRR[Leucine rich repeat]- and pyrin domain-containing protein 3) which functions as an intracellular sensor of D/M/L-AMPs resulting in activation of caspase-1, promotion of apoptosis, pyroptosis and generation of inflammatory cytokines. This review addresses the question of whether IP activation might be associated with male infertility situations.

**Results & conclusions:**

We conducted a systematic review of articles published in the Google Scholar, and PubMed databases through October 2021. It turns out that inflammasome activation and its consequences including cytokine storms, apoptosis and pyroptosis could be associated with the reduced sperm count as well as the structural and functional sperm defects recorded in several situations associated with male infertility suggesting that anti-inflammatory therapeutic strategies could be possibly considered to restore male fertility in future research.

## Résumé

**Contexte:**

L’infertilité liée à la varicocèle, aux infections, aux dysfonctionnements métaboliques, au stress oxydant et aux toxiques environnementaux est. également associée à des processus inflammatoires qui conduisent finalement à l’activation de la voie de l’inflammasome (IP). Cette voie est. classiquement activée par des DAMPs, MAMPs ou LAMPs, qui signifient respectivement Damage-, Microbe- ou Lifestyle-Associated Molecular Patterns. L’acteur le plus important dans l’activation de l’inflammasome est. la protéine NLRP3 (NOD[Nuclear Oligomerization Domain]-, LRR[Leucine Rich Repeat]- and pyrin domain-containing protein 3) qui fonctionne comme un capteur intracellulaire de D/M/L-AMPs résultant en l’activation de la caspase-1, la promotion de l’apoptose, la pyroptose et la génération de cytokines inflammatoires. Cette revue aborde la question de savoir si l’activation de l’inflammasome pourrait être associée à des situations d’infertilité masculine.

**Résultats et conclusions:**

Nous avons réalisé une revue systématique des articles publiés dans les bases de données Google Scholar, et PubMed jusqu’en octobre 2021. Il s’avère que l’activation de l’inflammasome et ses conséquences, y compris les “orages” de cytokines, l’apoptose et la pyroptose, pourraient être associées à la réduction du nombre de spermatozoïdes ainsi qu’aux défauts structurels et fonctionnels des spermatozoïdes enregistrés dans plusieurs situations associées à l’infertilité masculine, ce qui suggère que des stratégies thérapeutiques anti-inflammatoires pourraient être éventuellement envisagées pour restaurer la fertilité masculine dans les recherches futures.

## Introduction

A variety of pathogens such as viruses, bacteria, fungi, but also non-organic actors such as toxins whether exogenous or endogenous can endanger human health [1]. Classically, upon confrontation with a pathogen, the first line of defense that initiates the process of its elimination relies on innate immunity [2]. Highly conserved receptors, belonging to the Toll-like receptor (TLR) and pattern recognition receptors (PRR) families are present on the cell surface and serve as sensors to engage innate immunity. These receptors are also referred to as PAMPs or MAMPs (pathogen/microbial-associated molecular patterns) sensors, as well as DAMPs (damage-generated molecular patterns) or LAMPs (lifestyle-associated molecular patterns) sensors. When these sensors come into contact with their appropriate ligands, a downstream inflammatory pathway, called “inflammasome,” is triggered [3, 4].

**Fig. 1:**
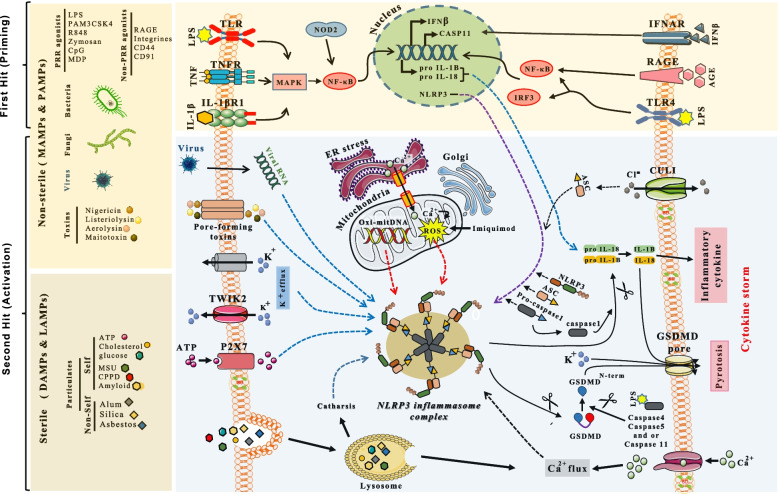
NLRP3 inflammasome complex, as part of innate immunity, is activated upon exposure to viruses, bacteria, fungi, but also non-organic actors. Exposure engaging innate immunity occur through sensors called Toll-like receptor (TLR) and pattern recognition receptors (PRR). Agents activating these sensors are referred to as PAMPs or MAMPs (pathogen/microbial-associated molecular patterns), DAMPs (damage-generated molecular patterns) or LAMPs (lifestyle-associated molecular patterns). Cell response to PAMPS/MAMPS is mediated through the formation of the NLRP3 inflammasome complex. Upon engagement of pathogen, the NLRP3 inflammasome complex is formed directly or through priming via NFќB. Priming induces formation of precursors of the NLRP3 inflammasome complex including NLRP3, pro-IL-1ß & pro-IL-18. Besides pathogens, particles, toxins and ATP release secondary to cell damage have the ability to activate the NLRP3 inflammasome. Activated NLRP3 inflammasome complex results in conversion of pro-caspase 1, pro-IL-1ß & pro-IL-18 to caspase1, IL-1ß and IL-18 leading to pyroptosis and inflammation

The inflammasome is determined by its PRR which, when activated via the recruitment of the adapter protein “apoptosis-associated speck-like protein containing caspase recruitment domain” (ASC), oligomerizes pro-caspase-1 to form active caspase-1. Of all the PRRs known to date, the most studied inflammasome is NLRP3 which plays a dominant role in both inflammation and antiviral responses [5, 6]. Unlike TLRs, NLRs (NOD-like receptors) are considered cytoplasmic intracellular sensors of DAMPs, MAMPs, PAMPs, and LAMPs (see Fig. 1). Once activated, caspase-1 recruited by ASC and NLRP3 cleaves the interleukin precursors pro-IL-1β and pro-IL-18 into their biologically active mature forms [7, 8]. It should be noted that other activation signals, such as ATP release, pore-forming toxins, viral RNA, K^+^ efflux, Ca^2+^ signaling, reactive oxygen species (ROS), transcriptional activator NFKB (Nuclear Factor Kappa B), mitochondrial dysfunction or lysosomal disruption (see Fig. 1) have been suggested to also activate NLRP3. However, only K^+^ efflux has been convincingly shown to activate NLRP3 while the other proposed activators remain controversial [5, 9]. Furthermore, in addition to active caspase-1, caspases 4, 5 and 11 can also cleave gasdermin D (GSDMD), an essential mediator of host defense resulting in pyroptosis [10], a type of cell death induced by cell membrane disruption (see Fig. 1). Finally, the hemichannels such as the hemichannel Pannexin-1 allowing ATP influx, was also shown to be involved in NLRP3 inflammasome activation through an interaction with the P2X7 purinergic receptor (see Fig. 1) leading to the release of interleukin-1ß and pyroptosis [11].

This inflammasome-mediated response is not limited to professional antigen-presenting cells (APCs) such as macrophages, as it has been shown that several epithelial cells can also behave in the same way. In the testis of mammalian, it has been shown that Sertoli cells, which function as non-professional tolerogenic APCs, have the ability to activate the inflammasome response via the TLR4/NOD/NFKB/Caspase-1/IL-1ß/IL-18 pathway [12]. Recently, it was reported that the NLRP3 inflammasome was activated in an experimental model of varicocele [13–16]. This suggests that activation of the NLRP3 inflammasome could be part or player of some infertility situations, as stated in previous reviews [17, 18], via an inflammatory cytokine storm and, it is likely that this could explain COVID-19-induced infertility as recently hypothesized [19]. In this context, the purpose of this review is to present recent findings regarding the activation of the NRLP3 inflammasome in the testis and its putative relationship with male infertility and how this could potentially be used to correct it.

## Methods

**Fig. 2:**
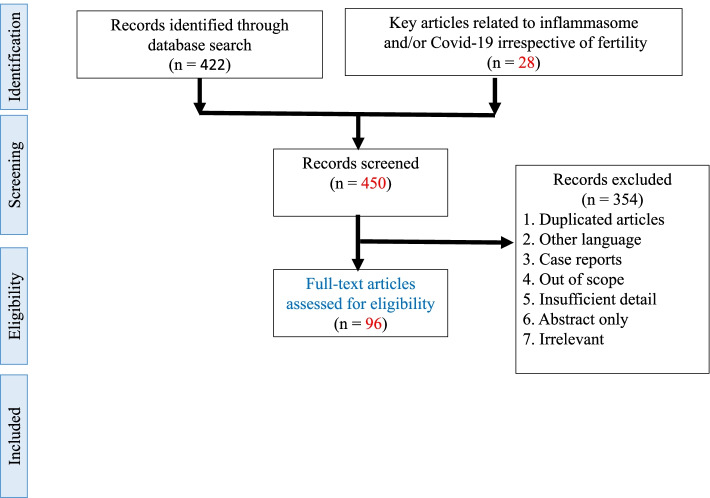
PRISMA flow diagram showing the literature data mining method used. Method of search: 1: Inflammasome, NLRP3, caspase-1, IL-1ß, IL-18 or ASC with male infertility, varicocele, semen, sperm, testis or epididymis. 2: COVID-19 with male infertility, varicocele, semen, sperm, testis or epididymis “and” main key words of inflammasome pathway (NLRP3, caspase-1, IL-1ß, IL-18 or ASC)

We conducted a systematic review of articles published in the Google Scholar, and PubMed databases through October 2021. Using the terms: inflammasome, NLRP3, caspase-1, IL-1ß, IL-18 or ASC with male infertility, varicocele, semen, sperm, testis or epididymis. We also searched for term COVID-19 with male infertility, varicocele, semen, sperm, testis or epididymis “and” aforementioned components of inflammasome pathway (NLRP3, caspase-1, IL-1ß, IL-18 or ASC). In addition, we used high quality review articles (*N* = 10) that were not related to male infertility to present the inflammatory pathway. We only considered English publications. Approximately 86% of the articles used for the current review paper were articles with high quality (Q1 and Q2) which were accepted in high impact journals. We have removed citations to other types of NLRP, except NLRP3. In addition, duplicate articles for each search with the above keywords, case report, abstract only, articles with insufficient details and out of range were also excluded from this study. Regarding the citation limitation, the main and latest relevant publications were mentioned (Fig. 2).

### NLPR3 in varicocele-related infertility

#### NLPR3 and varicocele

One of the well-documented etiologies of male infertility is varicocele (VCL), which has a frequency of approximately 4–22.6% in the general population, 21–41% in men with primary infertility and, 75–81% in men with secondary infertility [20]. VCL is mainly related to dilation of the pampiniform plexus causing reduced spermatozoa production, abnormalities in their morphology and motility. Increased testicular temperature associated with increased ROS generation are considered the main causative agents of VCL, leading to mitochondrial dysfunction, increased autophagy, apoptosis and endoplasmic reticulum (ER) stress in the testis of mammalian [21–24]. In addition to the increase in TUNEL-positive apoptotic cells, caspase-1 reactivity has also been recorded in rat experimental models of VCL [13]. Moreover, reports mention the release of pro-inflammatory cytokines, including IL-1α, IL-1ß and TNF-α (Tumor Necrosis Factor 1 alpha) from various testicular cells including germ cells, Leydig and Sertoli cells [25]. All of the above events are associated with activation of the inflammasome, suggesting that inflammasome activation may accompany VCL. This was recently confirmed as it was shown that the NLRP3 inflammasome pathway was indeed activated in the testis of VCL patients [14–16] although not up to the record of an increase in caspase-1 activity. Furthermore, the same authors reported an increase in ASC and downstream components of the inflammasome, including IL-18, in the semen of VCL patients compared to fertile control patients [16]. In addition, it has been shown that polymorph nuclear leucocytes which are elastase positive were also a source for IL-1ß and IL-18 [26]. In this regard, Camargo et al. (2021) demonstrated that unlike seminal IL-18, Caspase-1 and IL-1β were significantly higher in men with varicocele compared to control group. All three parameters were significantly reduced in men with varicocele when they underwent varicocelectomy surgery. They did not observe any significant difference in seminal ASC [27]. This may lead us to consider VCL as a typical inflammatory condition. It should be noted that, beside increased ROS (Reactive Oxygen Species) generation, VCL is also associated with elevated nitric oxide (NO) levels in human seminal plasma which was associated with low sperm motility [16, 28].

Although the role of Ca^2+^ signaling in the activation of NLRP3 inflammasome remains controversial [29] it is worth considering it. As Phospholipase-C (PLC) down-product IP3 (inositol triphosphate) is known to provoke the release of Ca^2+^ from the ER, it is hypothesized that PLC inhibition could partly inhibit NLRP3 inflammasome activation. Since PLC activation also promotes IL-1ß secretion [30] down-regulating PLC could be an interesting strategy to diminish the inflammasome activation and, consequently, the damaging effects it has on the VCL testis. In addition to PLC-mediated Ca^2+^ release, plasma membrane calcium sensory receptors, also known as store-operated Ca^2+^ entry (SOCE) channels, have been reported to induce NLRP3 activation and mitochondria dysfunction [9, 31].

#### Association between ROS and inflammasome in varicocele state

Considering the important role played by ROS in triggering as well as in the progression of VCL, several studies have addressed the question whether antioxidant supplementation could alleviate it and preserve male fertility. Lately, it was reported that “Gui-A-Cra” a commercially registered edible *Gryllus bimarculatus* insect powder has antioxidant, antimicrobial and anti-inflammatory activities [32, 33]. It has been proposed that it could be an appropriate supplemental that could diminish the symptoms associated with diabetes, liver disease, arthritis and VCL [33]. Concerning VCL, it was shown in animal VCL models that “Gui-A-Cra” could restore normal testis function by improving testosterone synthesis, reducing lipid peroxidation, reducing inflammation and enhancing the expression of AO enzymes [33]. Altogether, these combined effects were effective in reducing VCL-induced ER-stress as well as apoptosis [33]. The same group also showed earlier that, medicinal herbs having anti-oxidant, anti-inflammatory and anti-noniceptive properties could act similarly in a rat VCL model [34]. Similarly, Soni et al. (2018) [35] reported that a mixture of extracts from 3 medicinal herbs (commercially referred as MOTILISPERM™) could alleviate the effects of VCL on rat male fertility, mainly by its antioxidant action which limited VCL-induced testis ER stress. In a different report, resveratrol which is also known to have antioxidant, anti-inflammatory and anti-apoptotic activities, was shown to reduce VCL-induced testicular damage, apoptosis and inflammation in the rat VCL model [14]. Interestingly, in this last report, resveratrol was also shown to reduce the expression of the NLRP3 inflammasome players ASC and Caspase-1. Resveratrol also promoted the expression of the anti-apoptotic Bcl2 protein at the expense of the pro-apoptotic protein Bax.

In a different direction, it was proposed that testis VCL damaging effects in mammals could potentially be diminished by other means. For example, survivin and NAIP (neuronal apoptosis inhibitory protein) were shown to play significant roles in the rat, beside the inhibition of the executioner Caspase-3, therefore promoting survival. They were both shown to down-regulate inflammatory signaling pathways, thanks to their E3-ubiquitin ligase activities [36, 37]. Interestingly, both NAIP and survivin were shown to be significantly reduced in the rat VCL testis and, that their expression could be significantly increased by supplementation with PRDN (a polydeoxyribonucleotide extracted from trout or salmon sperm). PRDN is an agonist of adenosine A2A receptor (A2AR), a G protein-coupled receptor classically used for its tissue-repair activity based on its anti-inflammatory activity [38]. A2AR is also known to induce VEGF (vascular endothelial growth factor) expression in hypoxic condition as it is the case in VCL. However, it is also known to promote the release of IP3 which we have seen above could stimulate the activation of the NLRP3 inflammasome through Ca^2+^ release, an action that could be counteracted by the E3 ubiquitin-ligase activity of PRDN [36, 39]. More recently, Antonuccio et al. (2021) [13] reported that the addition of selenium to PRDN supplementation could further improve the testis status in VCL rats. Altogether these animal studies suggest that limiting the activation of the NLRP3-inflammasome could potentially represent a pertinent therapeutic strategy in order to reduce the damaging effect of VCL on the testis tissue. However, such therapeutic approaches need to be validated by clinical trials.

### Role of NLRP3 in male genital tract infection, inflammation and infertility

In the mammalian testis, Sertoli cells form the blood-testicular barrier (BTB), which is crucial in making the seminiferous tubules a site of immune privilege. Sertoli cells also secrete immunoregulatory factors that actively modulate the immune response. In addition to Sertoli cells, neighboring peritubular cells participate in this immune privilege situation [40]. A study showed that Sertoli cells and peritubular cells, in addition to expressing TLRs, also express key components required for NLRP3 inflammasome activation and that the level of NLRP3 expression may be related to infertility [41]. NLRP3 activation in peritubular cells was also associated with fibrotic thickening of seminiferous walls in situations of mast cell infiltration that constitute some cases of male infertility [42]. It should be noted that the testis in certain situations including infection, spinal cord injury, iatrogenic inflammation, may be in a state of oxidative stress leading to NLRP3 activation. In some of these cases, the increase in IL-1β can be prevented by administration of antioxidants or inhibitors of the NLRP3 pathway such as diacerein [43–45].

#### Role of NLRP3 in infection, inflammation and infertility

Apart from the studies mentioned above, it appears that a limited number of investigations have assessed NLRP3 in situations of male infertility. In contrast, many studies have measured cytokine levels, including IL-1β and Caspase 1 activation, as evidence of inflammation associated with male infertility situations (see for example: [17, 46, 47]. In an animal model of induced epididymitis, it was shown that the innate immune system was activated in a PAMP-specific manner leading to increased levels of cytokines IL-1ß and TNFα and that this is likely mediated by activation of the NLRP3 inflammasome [46, 48]. It is also noteworthy that noninfectious epididymitis can also occur secondary to germ cell injury through the induction of innate immune responses in epithelial epididymal cells associated with IL-1β [49].

In addition, IL-1ß and IL-18 were associated with elastase production by polymorphonuclear leukocytes both in idiopathic infertility and when infection was confirmed [26]. Sanocka et al. also showed that in case of infection, the cytokine expression pattern is different between fertile and infertile individuals who also show a different redox state [50]. This has led to the suggestion that in addition to monitoring leukocyte levels, which in itself has little prognostic value with respect to the patient’s fertility potential, an assessment of seminal pro-inflammatory cytokines and oxidative status might be useful. In the mammalian testis, IL-1ß seems to play a very important role [51–53]. Briefly, it has been shown that IL-1ß levels are higher in Leydig cells and interstitial cells compared to Sertoli cells and germ cells [54, 55]. It is also interesting to note that IL-1ß levels were found to be reduced in human testicular sections with impaired spermatogenesis [47, 56]. It has been suggested that this was somehow related to Leydig cell apoptosis [47, 57, 58]. In this context, homeostasis of the testicular IL-1ß pathway could regulate androgen production and consequently spermatogenesis [59]. This might be in line with the observation that IL-1ß or its receptor antagonist (IL-RA 2) polymorphism has been associated with defective spermatogenesis as well as defective sperm parameters [60], which are attributed to ROS-mediated damage [61]. Furthermore, it has been stated that IL1-RA, produced by Sertoli cells in response to IL-1ß, FSH or bacterial LPS, is involved in the modulation of spermatogenesis and fertility, which is reinforced by its lower expression in infertile men [62]. For these reasons, it was hypothesized that by inhibiting the NLRP3/Caspase-1/IL-1ß inflammasome pathway, it could be possible to reduce testicular inflammation and its consequences on gametes and male fertility [63, 64]. Despite the aforementioned reports highlighting the relationship between IL-1ß, TNFα and male fertility, there are reports that were unable to reveal such a relationship probably due to their small sample size [65, 66]. This hypothesis however proved to be correct in an experimental model of cadmium-induced testicular toxicity or testicular ischemia- reperfusion [67], in which diacerein, an inhibitor of Caspase-1 (also known as ICE-1: interleukin-1 converting enzyme), decreased both inflammasome activation and apoptotic pathways [68]. Inhibition of Caspase-1, in addition to limiting apoptosis could therefore be an effective way to limit inflammasome activation [69, 70]. Furthermore, it has been shown that in case of inflammation, toxins can aggravate the NLRP3 response and lead to germ cell apoptosis through the release of cytokines, notably IL-1ß and IL-18. This apoptosis can to some extent be prevented by administration of KLOTHO, a mediator of NLRP3 activation [71]. KLOTHO and FGF3, by regulating vitamin D, influence gonadal function and testicular mineral ion homeostasis which may improve sperm parameters. Melatonin, via its antioxidant, anti-proliferative and anti-inflammatory properties has also been shown elsewhere to be a potentially interesting molecule especially when it comes to reducing mast cell and testicular macrophage activation and the inflammation associated with these cells [72]. However, it should be noted that IL-1ß production and secretion is not solely dependent on the NLRP3 pathway, which may be a limitation of the therapeutic approach aimed solely at limiting inflammasome activation.

Beside IL-1ß, IL-18, another proinflammatory cytokine activated by Caspase-1, was found to be twice as represented in testes from infertile patients, particularly in varicocele and in individuals with genital *U. urealyticum* infections [25, 73]. Similarly, a significant change in IL-18 expression was reported in somatic and germ cells of normal and abnormal testicular sections from obstructive and non-obstructive azoospermic individuals [74, 75]. Elevated IL-18 levels in semen have been positively associated with a greater risk of pregnancy failure after IVF and ICSI, suggesting defective sperm function [76, 77]. This is not limited to IL-18, as a similar association between sperm quality and seminal cytokine content has also been reported for the pro-inflammatory cytokines IL-6 and TNFα [78]. It was concluded that pro-inflammatory cytokines such as IL-18 may have an important involvement in steroidogenesis and spermatogenesis.

#### Role of NLRP3 in spinal cord injury

Similarly, in the acute phase of spinal cord injury, disruption of the BTB results in secretion of IL-1ß by infiltrating macrophages, a potent pro-inflammatory cytokine that activates testicular endothelial signaling pathways, promoting neutrophil recruitment and ultimately leading to germ cell apoptosis [54]. This effect is partially maintained in case of chronic spinal cord injury [54]. Similarly, reports have shown that in infertile patients, a correlation could be established between impaired seminal parameters, oxidative stress and levels of pro-inflammatory cytokines, including IL-1ß and TNFα [55, 79]. In this regard, Zhang et al. (2013) demonstrated that mean of seminal ASC, caspase-1, IL-1b, and IL-18 were significantly higher in men with spinal cord injury compared to control group [80].

Activation of Pannexin-1 channels is one of the known pathways involved in the activation of the inflammatory pathway. Ibrahim et al. (2018) showed that administration of oral probenecid in men with spinal cord injury could improve percentage of sperm motility by interfering with these channels [81]. This group also demonstrated that incubation of semen samples of men with spinal cord injury with anti-ASC polyclonal antibody resulted in an increase in sperm motility compared to non-incubated samples due to neutralization of ASC [82].

#### Association NLRP3 inflammasome with age

It has been reported that testicular NLRP3 expression increases with age, a process called “inflammaging”, which is partially reversible by aromatase inhibitors. Aromatase inhibitors have also been shown to be useful in some cases of infertility associated with obesity-related systemic inflammation. However, it remains unclear whether in these high BMI infertile patients, the NLRP3 inflammasome is involved or not. Taken together, these data suggest that Sertoli cells and peritubular cells are major sites of NLRP3 expression in the testis of mammalian [83].

### Inflammasome and COVID-19

The SARS-COV-2 virus binds to the angiotensin-converting enzyme 2 (ACE2) receptor and enters the host cell. In addition to ACE2 receptor, the transmembrane protease, serine 2 (TMPRSS2) receptor also helps virus integration with the cell membrane by cutting SARS-COV spike proteins at the same time as ACE2 activity. ACE2 and TMPRSS2 receptors are expressed in most tissues of the body including the lung, kidney, intestine, heart, bladder, and tissues of the male genital tract. According to the literature, these are the tissues most targeted by Covid-19b [84]. In this regard, recent studies have demonstrated the high expression of ACE2 and TMPRSS2 receptors in spermatogonia, Sertoli and Leydig cells and in male accessory organs such as prostate, seminal vesicles and bulbourethral glands [85–88]. In an original study, when semen parameters and serum testosterone level were assessed after diagnosis of COVID-19, mean semen volume, sperm motility, normal sperm morphology and serum testosterone were found to be significantly decreased. It is hypothesized that this may be associated with the effect of SARS-COV-2 on Leydig cells via the ACE-2 receptors, resulting in reduced testosterone levels and decreased semen quality [89]. In addition, Yang et al. (2020) showed reduced Leydig cells, mild inflammation and altered seminiferous tubules in patients with COVID-19 [90].

As mentioned above, NLRP3 plays an important role in both inflammation and antiviral responses. In this context, an association has been anticipated between NLRP3, infertility, COVID-19 and its severity [91, 92]. The hypothesis is that differences in COVID-19 disease severity between individuals is explained by the level of their immune response in the early stages of the disease and the rate of virus entry into their body. In other words, the immune system of patients with mild symptoms had the ability to counter and eliminate the virus in the early stages of infection through a more effective innate and acquired immune responses. In an optimized response, this dual action is well balanced and leads to elimination of the viral agent. When the immune response is not optimal, the viral agent is not completely eliminated, leading to an increase in the NLRP3 inflammatory response due to the overexpression of inflammatory actors. This leads to what has been called a cytokine storm that results in severe tissue damage associated with organ failure and high mortality [91, 92]. Although NLRP3 activity, inflammation, and cytokine markers in patients with COVID-19 are well known aspects, the evaluation of NLRP3 in the human testis and sperm has not yet been reported. Few studies have only demonstrated in the human testis and sperm the activation of the downstream pathway of NLRP3, particularly with respect to proinflammatory cytokines and chemokines. Specifically, very recently, Hajizadeh-Maleki and Tartibian (2021) [93] reported overproduction of apoptotic (caspases 3, 8, and 9), inflammatory (IL-1β, IL-6, IL-8, IL-10, TGF-β, TNF-α, IFN-α, and IFN-γ) markers associated with oxidative stress in seminal plasma of COVID-19 men. COVID-19 could thus lead to a reduction in sperm production and semen quality parameters through activation of the apoptosis and inflammation pathways most likely secondary to inflammasome activation [93, 94]. A single case report very recently supports this assumption [95]. Confirmation of this scenario will require further investigation in larger number of COVID-19 male patients of childbearing age. A better understanding of the impact of COVID-19 on male fertility is definitely needed in order to appropriately counsel couples wishing to conceive about the potential collateral risks associated with COVID-19 infection. So that recently, it has been emphasized the importance of assessing the possible short and long-term consequences of infection in infertile couples with COVID-19 in the clinic [96].

## Conclusions

From this review of the literature, it appears that activation of the inflammasome and its associated pathways, including proinflammatory cytokine storms, oxidative stress, apoptosis, and pyroptosis, is likely to be part of a variety of inflammatory contexts leading to male infertility, from varicocele to male genital tract infection, including the very recent COVID-19 pandemic.

One of the highlights of this review is the study of the inflammasome pathway in the process of spermatogenesis with a particular focus on varicocele. Although many studies have used antioxidants to improve spermatogenesis in the treatment of male infertility, our main objective in this review was to show that, in addition to antioxidants, inflammasome inhibitors may have a role in the treatment of male infertility, particularly in cases of varicocele or COVID-19-related infertility due to the inflammatory storm associated with the latter. One of the strengths of this review proposal is to emphasize that inflammatory situations, whether from a varicocele situation or a viral infection, are both the conundrum and the master chief of male infertility situations. One of its major limitations is the paucity of reports in which the inflammatory status of patients, both systemic and testicular/seminal, has been examined in depth at a relevant time point.

## Data Availability

Not applicable.

## Abbreviations

AGE: Advanced Glycation End-products
Alum: Aluminium
ASC: Apoptosis-associated speck-like protein containing a caspase-recruitment domain
ATP: Adenosine triphosphate
Ca^2+^: Calcium
CASP11: Cysteine aspartase or cysteine-dependent aspartate-directed protease 11
Caspase 1: Cysteine aspartase or cysteine-dependent aspartate-directed protease 1
Caspase 11: Cysteine aspartase or cysteine-dependent aspartate-directed protease 11
Caspase 4: Cysteine aspartase or cysteine-dependent aspartate-directed protease 4
Caspase 5: Cysteine aspartase or cysteine-dependent aspartate-directed protease 5
CD44: Cell Surface Glycoprotein CD44
CD91: Cell Surface Glycoprotein CD91
Cl-: Chloride
CpG: 5′-C-phosphate-G-3′
CPPD: Calcium Pyrophosphate Dihydrate
CULI: Cullin1
DAMPs: Damage-Associated Molecular Patterns
ER stress: Endoplasmic reticulum stress
GSDMD pore: Gasdermin D pore
IFNAR: Interferon-Α/Β Receptor
IFNB: Interferon beta
IL-1ß: Interleukin 1-beta
IL-1ßR: Interleukin 1-beta receptor1
K^+^: Potassium
LAMPs: Lifestyle-Associated Molecular Patterns
LPS: Lipo-poly-saccharides
LRR: Leucine-rich repeat
MAMPs: Microbe-Associated Molecular Patterns
MAPK: Mitogen-Activated Protein Kinase
MDP: Muramyl dipeptide
MSU: Monosodium urate
NFKB: Nuclear factor kappa B
NLRP3: NLR family pyrin domain containing 3
NOD2: Nucleotide-binding and oligomerization domain 2
Oxi-mtDNA: Oxi-Mitochondrial DNA
PAM3CSK4: Pam3CysSerLys4
PAMPs: Pathogen-Associated Molecular Patterns
PRR: Pattern Recognition Receptors
P2X7: P2X purinoreceptor 7
RAGE: Receptor for Advanced Glycation End-products
ROS: Reactive oxygen species
Silica: Silica crystals
TLR: Toll-like receptors
TLR4: Toll-like receptor 4
TNF: Tumor necrosis factor
TNFR: Tumor necrosis factor Receptor
TWIK2: Two-pore domain K^+^ channel (K2P)
